# Epidemiology of Soil-Transmitted Helminth Infections among Primary School Children in the States of Chhattisgarh, Telangana, and Tripura, India, 2015–2016

**DOI:** 10.4269/ajtmh.21-1185

**Published:** 2022-05-16

**Authors:** Sandipan Ganguly, Sharad Barkataki, Prerna Sanga, K. Boopathi, Kaliaperumal Kanagasabai, Shanmugasundaram Devika, Sumallya Karmakar, Punam Chowdhury, Rituparna Sarkar, Dibyendu Raj, Leo James, Shanta Dutta, Suzy J. Campbell, Manoj Murhekar

**Affiliations:** ^1^ ICMR-National Institute of Cholera and Enteric Diseases, Kolkata, India;; ^2^ Deworm the World Initiative, Evidence Action, New Delhi, India;; ^3^ GFK Mode, Mumbai, India;; ^4^ ICMR-National Institute of Epidemiology, Chennai, India;; ^5^ Deworm the World Initiative, Evidence Action, Brisbane, Australia

## Abstract

Soil-transmitted helminth (STH) infections are highly prevalent in many developing countries, affecting the poorest and most deprived communities. We conducted school-based surveys among children studying in first to fifth standard in government schools in the Indian States of Chhattisgarh, Telangana, and Tripura to estimate the prevalence and intensity of STH infections during November 2015 and January 2016. We adopted a two-stage cluster sampling design, with a random selection of districts within each agro-climatic zone in the first stage. In the second stage, government primary schools were selected by probability proportional to size method from the selected districts. We collected information about demographic details, water, sanitation, and hygiene (WASH) characteristics and stool samples from the school children. Stool samples were tested using Kato-Katz method. Stool samples from 3,313 school children (Chhattisgarh: 1,442, Telangana: 1,443, and Tripura: 428) were examined. The overall prevalence of any STH infection was 80.2% (95% confidence interval [CI]: 73.3–85.7) in Chhattisgarh, 60.7% (95% CI: 53.8–67.2) in Telangana, and 59.8% (95% CI: 49.0–69.7) in Tripura. *Ascaris lumbricoides* was the most prevalent STH infection in all three states. Most of the STH infections were of light intensity. Our study findings indicate that STH infections were highly prevalent among the school children in Chhattisgarh, Telangana, and Tripura, indicating the need for strengthening STH control program in these states. The prevalence estimates from the survey would serve as a baseline for documenting the impact of the National Deworming Day programs in these states.

## INTRODUCTION

*Ascaris lumbricoides*, *Trichuris trichiura*, and hookworms *Necator americanus* and *Ancylostoma duodenale* are the commonest soil-transmitted helminths (STHs), accounting for loss of nearly two million disability-adjusted life years.^1^ STH are some of the most common infections contributing heavily to intestinal damage, anemia, and impaired physical growth and cognitive performance in children.^2^ Periodic anthelminthic treatment reduces the number of individuals with heavy infections; reduces environmental contamination and risk of infection for other people; reduces micronutrient loss (e.g., iron loss through intestinal bleeding in hookworm infection); and improves nutritional status, cognitive function, and learning ability.^3^ School-based deworming programs are considered as simple, safe, cost-effective, and scalable interventions to reach high-risk populations.^4^^,^^5^

In 2014, the WHO estimated that by number, India has the highest burden of STH infections in the world, with 223 million children aged 1–14 years at risk.^6^ Although the published studies indicate heterogenous burden of STH in the country, with prevalence ranging from 0.6% to 91%, with *A. lumbricoides* as the predominant species,^7^ large-scale surveys estimating the prevalence at the state level are limited. Such estimates are required to determine the frequency of preventive chemotherapy.^8^ Results of two multi-site state-wide surveys in Bihar (*N* = 1,279, conducted in 2011) and Uttar Pradesh (*N* = 6,421, conducted in 2015) indicated high STH prevalence, ranging between 68% and 76%.^3^^,^^9^ Besides these two studies, information about STH prevalence and intensity data were not available from other Indian states.

Ad hoc deworming had been conducted in some states in India, often as part of other initiatives, particularly annual mass drug administration (MDA) using a single dose of 400 mg of albendazole in districts where lymphatic filariasis (LF) was endemic, and provision of deworming tablets within the Weekly Iron and Folic Acid Supplementation (WIFS) Program in some areas.^10^ Although the reported coverage of MDA was generally higher (more than 80%),^11^ there is no information about validated coverage of LF-MDA, and more importantly compliance. In 2014, the Government of India made concerted efforts to scale up STH control activities to meet the WHO global commitment to overcome the impact of neglected tropical diseases.^12^ As a key step toward this, cross-sectional, cluster-sampled, school-based surveys were conducted in several States, including Chhattisgarh and Telangana (late 2015), and Tripura (early 2016)—totalling a geographic area of 314,568 km and population of over 67.5 million people.^13^ The objective of these surveys was to estimate the prevalence and intensity of STH infections among school-aged children studying in first to fifth standard in these states. The secondary objectives were to estimate the prevalence according to age group, sex, and by agro-climatic zone and to develop geospatial predictive maps of STH prevalence, encompassing the environmental diversity of each state.

## MATERIALS AND METHODS

### Ethics, consent, and permission.

The Institutional Ethics Committee of the Indian Council of Medical Research—National Institute of Epidemiology, Chennai, approved study protocols for each state. Written informed consent from parents of all students assenting to participate in the study was obtained prior to the interviews. In all states, the surveys were conducted prior to MDA. Permission to conduct the survey in schools was obtained from the Department of Health as well as the Department of Education of the respective states.

### Study setting.

**Figure 1.**
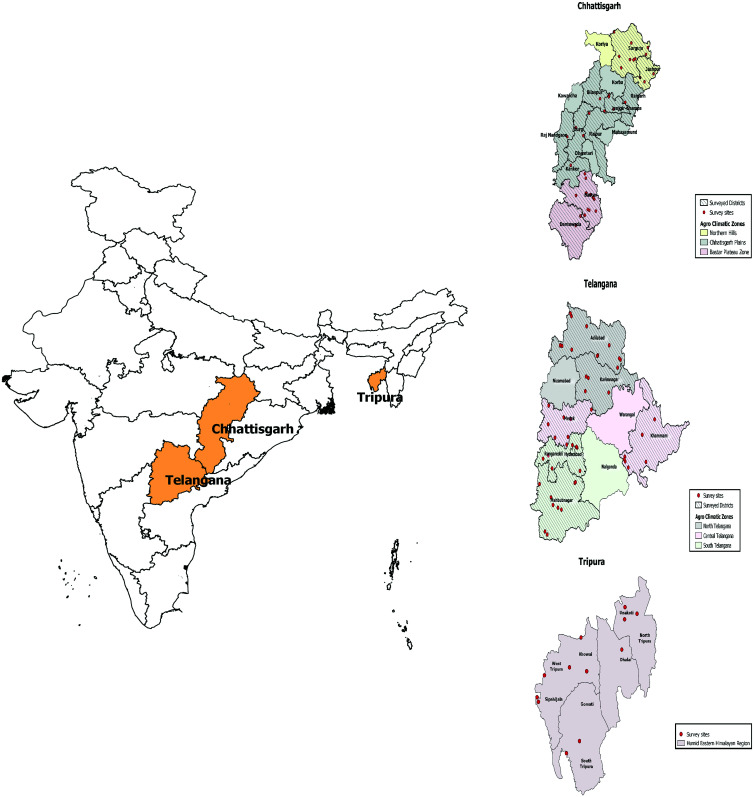
Map of India showing agro-climatic zones and survey locations for the three states. This figure appears in color at www.ajtmh.org.

As per the 2011 census, the total population of Chhattisgarh was 25,540,196, Telangana, 35,193,978, and Tripura 3,671,032, respectively.^13^ The WHO recommends sampling on the basis of agro-climatic zones (regions similar in environmental and climatic conditions), in proportion to the population of these zones.^8^ Based on soil type, average annual rainfall, and temperature, Chhattisgarh is divided into three agro-climatic zones: the Bastar plateau consisting of six districts, Chhattisgarh plains consisting of 16 districts, and hilly areas consisting of five districts.^14^ Telangana is divided into three agro-climatic zones—the north zone consisting of three districts, central zone consisting of three districts, and south zone consisting of four districts.^15^ Tripura state, with eight districts, has only one agro-climatic zone^16^ (Figure 1).

### Study design and population.

To develop state-wide estimates of STH prevalence and intensity, we followed the WHO-recommended sentinel site approach.^8^ We considered schools as sentinel sites and from each of the selected school, stool specimens from approximately 50 children were investigated for the presence of parasite eggs. The cross-sectional surveys were conducted among primary school children in classes one to five studying in government schools.

A sample size of 492 (rounded to 500) children was required per agro-climatic zone, assuming prevalence of any STH of 20% (chosen as preventive chemotherapy is recommended if STH prevalence is > 20%), absolute precision of 5%, a confidence level of 95%, and design effect of 2. Thus, the total sample size required to estimate the prevalence of STH for each of Chhattisgarh and Telangana states was 1,500, and 500 for Tripura. With 50 children to be sampled from each school, a minimum of 10 schools were needed from each zone. Incorporating a nonresponse of 20%, 60 children from each school were enrolled.

### Sampling procedure.

A two-stage sampling procedure was followed for selecting schools. In the first stage, districts were randomly selected from each agro-climatic zone. In the second stage, all the government primary schools of the districts selected from each zone were line-listed. Ten schools from each zone were then selected by probability proportional to size from the list of schools in the selected districts. School lists were then examined to determine the student population in each school. In cases where the population of the selected school was less than 60, one nearby additional school was selected to allow for a total of 60 children to be sampled from the area. This additional school was either in the same village/town or in the nearby village/town of the same block (sub-district). Children were then selected from enumerated class lists.

### Data collection.

The surveys were conducted during November 2015–January 2016. Trained survey teams visited the assigned schools, informed the head of the school about the survey objectives, and obtained permission to conduct the survey. The field teams then enrolled 60 children from the selected class, collected their contact details, entered school characteristics, and recorded the GPS location of the school. The survey teams then visited the houses of enrolled children, explained the purpose of the study, and obtained written informed consent from parents/head of the household. The respondents (mainly the mothers) of the selected children were interviewed to collect information about socio-demographic details, house type, source of drinking water, presence of toilet facilities and their use, hand washing practices, history of deworming, and regular use of footwear. Stool specimen containers were provided and the method of stool collection was explained to the children as well as the respondents. All questionnaires were administered using computer-assisted personal interview devices.

### Collection, transport, and processing of stool samples.

Single stool samples were collected in the mornings from the houses of the selected children, and transported in cool boxes to the field laboratory within 3–4 hours of sample collection. In the laboratory, samples were kept in cool boxes with ice packs until processed. The field laboratory was set up in the nearest district hospital.

Stool samples were processed using the WHO-recommended Kato-Katz method (Vestergaard Frandsen, New Delhi, India), following the manufacturer’s instructions.^8^ For each sample, two slides were prepared and independently examined by two parasitologists. The egg counts were used to estimate the prevalence and intensity of STH infections, with intensity of infection measured as eggs per gram of stool, and classified into light, moderate, and heavy infections according to the WHO guidelines.^8^ For quality control, 10% of the slides were re-read by exchange between the parasitologists. An independent expert reviewed the laboratory and survey procedures.

### Data analysis.

Parasitological and questionnaire data were imported into STATA (version 14) and merged to form a dataset linked by child unique identifier. Data were analyzed using the survey data analysis module of STATA to estimate the prevalence and intensity of STH infections in each state after adjusting for clustering. The prevalence was also estimated separately for age group, sex, and different agro-climatic zones. A χ^2^ test was used to compare proportions. A geographic information system (GIS)-based spatial interpolation—inverse distance weighting method was used to generate predictive maps of the prevalence of STH in the state using the observed prevalence data from the surveyed districts. The locations of the schools surveyed along with the unweighted prevalence of STH infections were integrated into GIS, and ArcGIS v. 10 (ESRI, Redlands, CA) was used for mapping.

## RESULTS

The survey was conducted in 95 schools from 26 districts in three states (Table 1). Most schools (*n* = 81, 85.3%) were from rural areas, 79 (83.2%) had a source of drinking water, and 82 (86.3%) had toilet facilities within the school premises. A total of 4,099 children were enrolled and stool samples were collected from 3,579 (87.3%). A total of 266 samples (7.4%) were rejected because of inadequate quantity, or sample being mixed with urine/water/soil. Therefore, against the target sample size of 3,500, stool samples from 3,313 (94.7%) children were analyzed to estimate the prevalence of STH in the three states.

**Table 1:** Sampling details and school characteristics

	Chhattisgarh	Telangana	Tripura	Total all states
Agro-climatic zones	3	3	1	
Required sample size*	1,500	1,500	500	3,500
Total districts in the state	27	10	8	45
Number of districts surveyed	13	6	7	26
Number of schools surveyed	40	43	12	95
Number of schools from rural areas	32	39	10	81
Number of schools with toilet facilities	37	34	11	82
Number of schools with a source of drinking water	36	31	12	79
Number of children enrolled	1,734	1,822	543	4,099
Stool samples collected, *n* (% of enrolled children)	1,597 (92.1)	1,535 (84.3)	447 (82.3)	3,579 (87.3)
Stool samples examined, *n* (% of samples collected)	1,442 (90.3)	1,443 (94.0)	428 (95.7)	3,313 (92.6)

* 500 per agro-climatic zone.

### General characteristics of children surveyed.

The mean age of children surveyed was 8.6 years (SD: 1.8) in Chhattisgarh, 8.2 years (SD: 1.9) in Telangana, and 8.6 years (SD: 1.3) in Tripura. Nearly half of the surveyed children (46.4%, *n* = 1,538) were males, and 36.6% (Telangana) to 55.6% (Tripura) belonged to the Scheduled Caste/Tribe (the officially designated groups of disadvantaged people in India). Fathers of 29.3% (*n* = 423) of children from Chhattisgarh, 48.0% (*n* = 692) of children from Telangana, and 6.5% (*n* = 28) of children from Tripura had no formal education. About a third (35.9%, *n* = 518) of the households in Chhattisgarh, 42.8% (*n* = 183) of households in Tripura, and 60.1% (*n* = 867) of households in Telangana reported having a piped water supply. Nearly two-thirds of the children in Chhattisgarh (*n* = 1,005, 69.7%) and Telangana (*n* = 937, 64.9%), but very few children in Tripura (15, 3.5%), reported practicing open defecation (Table 2).

**Table 2:** Sociodemographic characteristics of children surveyed, Chhattisgarh, Telangana, and Tripura, 2015–2016

		Chhattisgarh (*N* = 1,442)		Telangana (*N* = 1,443)		Tripura (*N* = 428)	
		*n*	%	*n*	%	*n*	%
Age group (years)							
5–7		431	29.9	555	38.5	92	21.5
8–10		766	53.1	741	51.4	310	72.4
> 10		245	17.0	147	10.2	26	6.1
Median age (IQR) (years)		9 (7–10)		8 (7–10)		9 (8–10)	
Sex		655	45.4	653	45.3	230	53.7
Male							
Family size							
≤ 5		700	48.5	1,096	76.0	350	81.8
6–10		693	48.1	332	23.0	74	17.3
> 10		49	3.4	15	1.0	4	0.9
Presence of BPL* card with the family		1,212	84.1	1,396	96.7	224	52.3
Ownership of house								
Own house		1,298	90.0	1,252	86.8	394	92.1
Rented		128	8.9	179	12.4	12	2.8
Other†		16	1.1	12	0.8	22	5.1
Caste‡								
General/other backward class		668	46.3	915	63.4	190	44.4
Scheduled caste/tribe		774	53.7	528	36.6	238	55.6
Religion								
Hindu		1,397	96.9	1,294	89.7	347	81.1
Muslim		13	0.9	92	6.4	72	16.8
Christian		21	1.5	57	4.0	7	1.6
Other		11	0.8	0	–	2	0.5
Education of father								
No formal education		423	29.3	692	48.0	28	6.5
Primary/middle school		737	51.1	603	41.8	288	67.3
Secondary school or above		282	19.6	148	10.3	112	26.2
Education of mother								
No formal education		582	40.4	813	56.3	47	11.0
Primary/middle school		710	49.2	559	38.7	292	68.2
Secondary school or above		150	10.4	71	4.9	89	20.8
Occupation of father								
Wage laborer		397	27.5	705	48.9	170	39.7
Agriculture/animal husbandry/allied activity		701	48.6	512	35.4	122	28.5
Self-used/service		312	21.6	213	14.8	119	27.8
Other		32	2.2	13	0.9	17	4.0
Occupation of mother								
Home maker		545	37.8	175	12.1	377	88.1
Wage laborer		320	22.2	746	51.7	11	2.6
Agriculture/animal husbandry/allied activity		498	34.5	362	25.1	23	5.4
Self-used/service		68	4.7	152	10.5	14	3.3
Other		11	0.8	8	0.6	3	0.7
Type of house§								
Kuccha wall and roof		828	57.4	269	18.6	293	68.5
Semi pucca		428	29.7	703	48.7	98	22.9
Pucca (pucca wall and roof)		186	12.9	471	32.6	37	8.6
Source of drinking water								
Public tap/piped water		518	35.9	867	60.1	183	42.8
Tube well/bore well		783	54.3	432	29.9	158	36.9
Unprotected dug well or spring		70	4.9	14	1.0	30	7.0
Protected dug well		44	3.1	44	3.0	45	10.5
Other		27	1.9	86	6.0	12	2.8
Place of defecation								
Open field		1,005	69.7	937	64.9	15	3.5
Own latrine/community latrine		437	30.3	506	35.1	413	96.5
Hand washing by the child after defecation								
Does not wash/wash with water		275	19.1	899	62.3	118	27.6
Wash with ash/mud		242	16.8	19	1.3	41	9.6
Wash with soap		925	64.2	525	36.4	269	62.9
Practice of using footwear regularly		1,044	72.4	896	62.1	383	89.5
Child reported taking medicine‖ for filariasis prevention within last year		428	29.7	261	18.1	61	14.3

* Below poverty line (BPL) cards are ration cards issued by the government to households living below the poverty line.

† Other household ownership status is primarily government-allotted housing.

‡ The main caste categories are Scheduled Caste and Scheduled Tribe (which have historically been more disadvantaged), as well as other backward class and general caste.

§ Kuccha houses are made from mud, thatch, or other low-quality materials. Pucca houses are made with high-quality materials throughout, including the floor, roof, and exterior walls; they are designed to be solid and permanent.

‖ Diethylcarbamazine distributed through lymphatic filariasis MDA program.

### Prevalence and intensity of STH infections.

**Figure 2.**
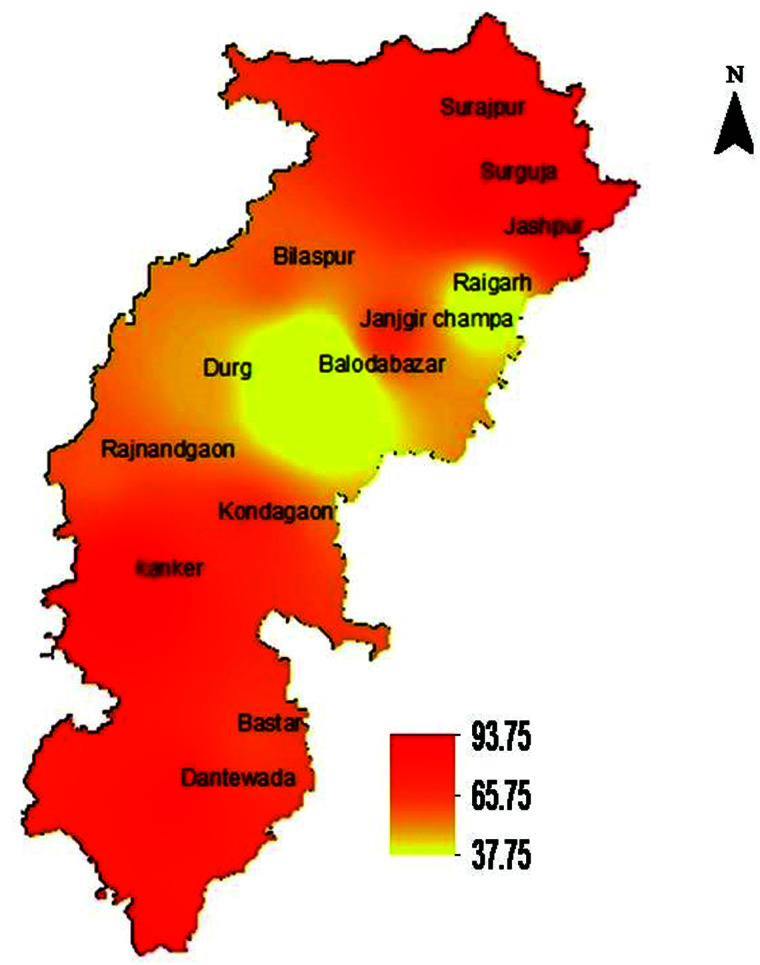
Predicted prevalence map of soil-transmitted helminth (STH), Chhattisgarh. This figure appears in color at www.ajtmh.org.

In Chhattisgarh, 1,157 of the 1,442 children whose stool samples were examined had one or more STH infections, with a prevalence of 80.2% (95% confidence interval [CI]: 73.3–85.7) after adjustment for clustering. The prevalence exceeded almost 70% in all the three agro-climatic zones. The STH prevalence in hilly areas was highest at 91.5% (95% CI: 87.5–94.3; Table 3, Figure 2). *Ascaris lumbricoides* was the most common STH infection in all the three agro-climatic zones (73.6%, 95% CI: 65.4–80.4). The prevalence of hookworm infection was 20.3% (95% CI: 13.3–29.6), and only two positive samples for *T. trichiura* were identified during the survey. Of the 1,157 children with any STH infections, 959 (82.9%) had one infection, whereas 198 (17.1%) had two STH infections. The prevalence of any STH in the state was 79.8% (95% CI: 71.3–86.3) among children aged 5–7 years, 79.6% (95% CI: 71.6–85.9) among children aged 8–10 years, and 82.9% (95% CI: 68.6–91.5) among children aged above 10 years. The prevalence of any STH was not statistically different by age group (*P* = 0.7936) or sex (*P* = 0.8522) (Table 4). Most infections of any helminth species were of light infection intensity, whereas 1.5% (*n* = 21) *A. lumbricoides* infections and 0.5% (*n* = 7) hookworm infections were of moderate intensity, and 0.4% (*n* = 6) hookworm infections of heavy intensity (Table 5). Both *T. trichiura* infections were of light infection intensity.

**Table 3:** Prevalence of STH in different agro-climatic zones, Chhattisgarh, Telangana, and Tripura, 2015–2016

State	Agro-climatic zone	No. examined	*Ascaris lumbricoides*		*Trichuris trichiura*		Hookworm		Any STH	
			No. positive	% (95% CI)	No. positive	% (95% CI)	No. positive	% (95% CI)	No. positive	% (95% CI)
Chhattisgarh										
Bastar plateau		465	289	62.2 (48.9–73.8)	2	0.43 (0.057–3.2)	162	34.8 (20.6–52.4)	371	79.8 (72.2–85.7)
Chhattisgarh plains		482	324	67.2 (51.8–79.7)	0	–	22	4.6 (1.7–11.8)	333	69.1 (54.3–80.8)
Hilly areas		495	448	90.5 (86.4–93.5)	0	–	108	21.8 (12.4–35.6)	453	91.5 (87.5–94.3)
Overall		1,442	1,061	73.6 (65.4–80.4)	2	0.14 (0.02–1.1)	292	20.3 (13.3–29.6)	1,157	80.2 (73.3–85.7)
Telangana										
North		490	346	70.6 (61.9–78.1)	0	–	39	8.0 (4.7–13.1)	354	72.2 (63.3–79.7)
Central		460	229	49.8 (39.5–60.1)	1	0.2 (0.03–1.6)	5	1.1 (0.4–2.6)	231	50.2 (39.8–60.6)
South		493	286	58.0 (45.5–69.6)	3	0.6 (0.2–1.7)	12	2.4 (1.1–5.3)	291	59.0 (46.6–70.4)
Overall		1,443	861	59.7 (52.9–66.1)	4	0.3 (0.1–0.7)	56	3.9 (2.5–6.1)	876	60.7 (53.8–67.2)
Tripura		428	219	51.2 (37.1–65.0)	36	8.4 (2.4–25.3)	24	5.6 (1.8–16.3)	256	59.8 (49.0–69.7)

**Table 4:** Prevalence of any STH among primary school children by age group and sex, Chhattisgarh, Telangana, and Tripura, 2015–2016

	Chhattisgarh			Telangana			Tripura		
	No. tested	No. positive for any STH	Prevalence (95% CI)	No. tested	No. positive for any STH	Prevalence (95% CI)	No. tested	No. positive for any STH	Prevalence (95% CI)
Age groups (years)									
5–7	431	344	79.8 (71.3–86.3)	555	342	61.6 (52.7–69.8)	92	50	54.4 (39.3–68.7)
8–10	766	610	79.6 (71.6–85.9)	741	443	59.8 (52.1–67.0)	310	193	62.3 (52.0–71.5)
> 10	245	203	82.9 (68.6–91.5)	147	91	61.9 (52.3–70.6)	26	13	50.0 (23.2–76.8)
Sex									
Male	655	527	80.5 (72.9–86.3)	653	410	62.8 (54.2–70.6)	230	143	62.2 (51.3–71.9)
Female	787	630	80.1 (72.7–5.8)	790	466	59.0 (52.3–65.2)	198	113	57.1 (44.0–69.2)

**Table 5:** Intensity of STH infections in Chhattisgarh, Telangana, and Tripura, 2015–2016

State	Intensity of STH infection	*Ascaris lumbricoides*		Hookworm		*Trichuris trichiura*	
		Number	% (95% CI)	Number	% (95% CI)	Number	% (95% CI)
Chhattisgarh	No infection	381	26.4 (19.6–34.7)	1,150	79.8 (70.4–86.7)	1,440	99.9 (98.94–99.98)
	Light	1,040	72.1 (64.4–78.8)	279	19.4 (12.8–28.2)	2	0.1 (0.02–1.06)
	Moderate	21	1.5 (0.8–2.6)	7	0.5 (0.2–1.1)	–	–
	Heavy	–	–	6	0.4 (0.2–1.0)	–	–
	Mean eggs per gram of stool	751		407		24	
Telangana	No infection	582	40.3 (33.9–47.1)	1,387	96.1 (94.0–97.5)	1,439	99.7 (99.3–99.9)
	Light	856	59.3 (52.6–65.8)	56	3.9 (2.5–6.1)	3	0.2 (0.1–0.6)
	Moderate	5	0.3 (0.12–0.96)	–	–	1	0.1 (0.009–0.5)
	Heavy	–	–	–	–	–	–
	Mean eggs per gram of stool	294		113		285	
Tripura	No infection	209	48.8 (35.0–62.9)	404	94.4 (83.8–98.2)	392	91.6 (74.7–97.6)
	Light	218	50.9 (36.9–64.8)	24	5.6 (1.8–16.3)	26	6.1 (2.3–15.0)
	Moderate	1	0.2 (0.02–2.3)	–	–	7	1.6 (0.2–13.0)
	Heavy	–	–	–	–	3	0.7 (0.1–5.9)
	Mean eggs per gram of stool	343		145		2,113	

**Figure 3.**
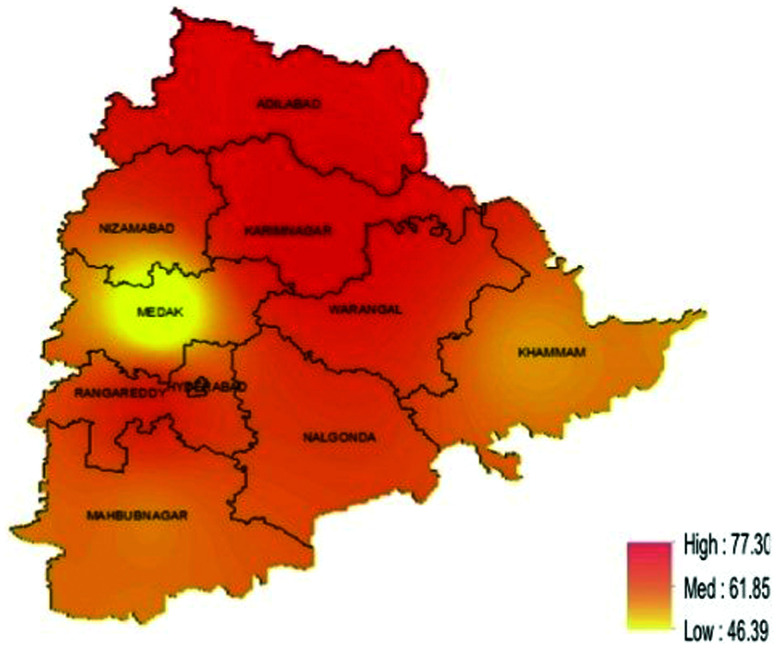
Predicted prevalence map of soil-transmitted helminth (STH), Telangana. This figure appears in color at www.ajtmh.org.

In Telangana, 876 of the 1,443 school children surveyed had any STH infection with a prevalence of 60.7% (95% CI: 53.8–67.2). The prevalence ranged between 50% and 72% in the three agro-climatic zones, with the highest prevalence in the northern region (Figure 3). *Ascaris lumbricoides* was the most common STH infection in all zones, with a prevalence of 59.7% (95% CI: 52.9–66.1), followed by hookworm (3.9%, 95% CI: 2.5–6.1) (Table 3). Four stool samples were positive for *T. trichiura* (0.3%, 95% CI: 0.1–0.7). Most (*n* = 831, 94.9%) of the STH-infected children had a single infection, whereas 45 (5.1%) had two infections. By age group, the prevalence of any STH in Telangana was 61.6% (95% CI: 52.7–69.8) among children aged 5–7 years, 59.8% (95% CI: 52.1–67.0) among children aged 8–10 years, and 61.9% (95% CI: 52.3–70.6) among children aged above 10 years. The prevalence of any STH did not differ by age group (*P* = 0.8075) or sex (*P* = 0.1964) (Table 4). Majority of the infections were of light infection intensity (Table 5).

**Figure 4.**
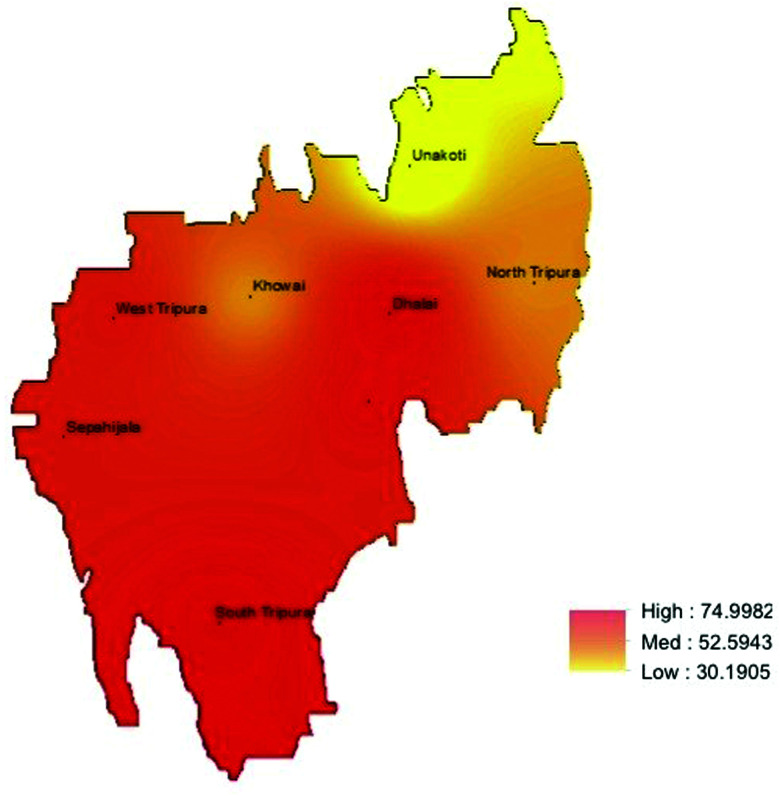
Predicted prevalence map of soil-transmitted helminth (STH), Tripura. This figure appears in color at www.ajtmh.org.

In Tripura, 256 of the 428 children surveyed had any STH infection, with a prevalence of 59.8% (95% CI: 49.0–69.7). *Ascaris lumbricoides* was the commonest STH infection (51.2%, 95% CI: 37.1–65.0) (Table 3). STH prevalence was higher in the southern region of the state (Figure 4). Interestingly, in Tripura, *T. trichiura* was the second most prevalent STH with prevalence of 8.4% (95% CI: 2.4–25.3), followed by hookworm (5.6%, 95% CI: 1.8–16.3). Most (*n* = 235, 91.8%) children had a single infection, whereas 19 (7.4%) and 2 (0.8%) were infected with two and three helminths, respectively. The prevalence of any STH in Tripura was 54.4% (95% CI: 39.3–68.7) among children aged 5–7 years, 62.3% (95% CI: 52.0–71.5) among children aged 8–10 years, and 50.0% (95% CI: 23.2–76.8) among children aged above 10 years. The prevalence of any STH did not differ by age group (*P* = 0.2932) or sex (*P* = 0.3061) (Table 4). Most infections were of light infection intensity (Table 5).

In all the three states, we did not find any association between the behavioral practices and STH prevalence (data not shown).

## DISCUSSION

This paper highlights the STH prevalence and intensity in the Indian states of Chhattisgarh, Telangana, and Tripura. Findings indicate that the overall prevalence of any STH infection among the children studying in government primary schools in all the three states was very high, with about 60–80% of school children infected with one or more STH. The WHO recommends annual treatment in areas where STH prevalence is between 20% and 50%, and biannual treatment in areas with prevalence rates of over 50%.^8^ Therefore, according to these guidelines, these states need two rounds of deworming annually to achieve optimal reductions in the prevalence. In 2015, the Government of India launched the National Deworming Day (NDD) targeting children aged 1–19 years, in government schools, government-aided schools, private schools, and *anganwadis* (preschools). The results of our surveys will serve as a baseline to measure the impact of the NDD program in these states over time.

Among the three states, STH prevalence was highest in Chhattisgarh, with 70–90% of children from each of the different agro-climatic zones infected with STH. In all the three states, STH prevalence was high in both sexes and across all age groups; with 50–80% of children aged 5–7 years positive for any STH. High STH prevalence in this age group indicates that STH infections are acquired during the preschool and early primary school years. The predominance of *A. lumbricoides* in all the three states is consistent with the findings of previous school-based surveys conducted in the Indian states of Bihar (51.9%) and Uttar Pradesh (69.6%).^3^^,^^9^ The prevalence of hookworm infection was higher in Chhattisgarh as compared with Telangana and Tripura. Earlier surveys conducted in Bihar and Uttar Pradesh following the similar methodology indicated the prevalence of hookworm infection of 42% and 23%, respectively. The practice of not wearing foot wear, which is a known risk factor for hookworm infection,^17^^,^^18^ was prevalent among school children in Chhattisgarh (27.6%) and Telangana (37.9%) as compared with Tripura (10.5%), suggesting that the differences in the prevalence could not be solely on account of this behavior. The delay in processing of stool samples lead to faster disintegration of hookworm eggs compared with *Ascaris* and *Trichuris*.^19^ Since field transportation and laboratory preparedness were carefully monitored during the surveys, our findings suggest heterogeneity of hookworm as well as STH distribution across different states.

Although deworming programs are often school-based because of the generally large burden of morbidity in school-age children, practical ease of accessing the children, as well as cost-effectiveness of school-based deworming, the age-specific prevalence of STH in the three states emphasizes the importance of deworming in preschool children as well. Children of youngest ages have the greatest growth velocities, and hence impact of STH in these formative years is expected to be highly deleterious.^20^ The target age group of 1–19 years for the NDD in India is expected to reduce the overall prevalence of STH in preschool as well as school-aged children.

Although the overall STH prevalence was high, a marked feature of all the three state surveys was generally the light intensity of infection. Overall, across any species, only 51 children, or 1.5% of those surveyed, had moderate-to-heavy intensity of infection. Given these states had not received prior large-scale MDA, these findings could possibly indicate that morbidity because of STH in these areas may be low, as it is the heavy-intensity infections that are linked to higher morbidity in populations.^4^^,^^21^^,^^22^ However, it is increasingly becoming recognized that light-intensity infections can also contribute considerably to morbidity in populations. Some studies have shown that even asymptomatic or light-intensity hookworm infections contribute to anemia in preschool and school-aged children.^23^^–^^25^ The clinical appearance of STH infection often lacks specific symptoms, which may not be recognized by the infected person, even when contributing to significant health damage.^26^ With prevalence recorded as high as 90% in some areas, a clear conclusion is that STH are a public health problem in these states of India.

The study had certain limitations. First, the survey was conducted in government schools, with no private schools surveyed. Hence, the actual prevalence of STH infections among school children in these states might have been overestimated. Second, although the WHO recommends use of the Kato-Katz method for STH prevalence surveys, the technique is known to have low sensitivity as a diagnostic test especially for light-intensity infections. Future surveys would benefit from alternative diagnostic techniques such as real-time polymerase chain reaction, if these can be cost-effectively implemented on a large scale.

There are particular strengths to this study. This is one of the very few epidemiological studies of STH prevalence and infection intensity, at state-wide scale in India. Given the large population size of these states, this is an important addition to the literature internationally as well as nationally. The use of standard STH survey methodology provided one of the most comprehensive large-scale assessments of STH epidemiology thus far available within India.

## CONCLUSIONS

These state-wide surveys provide evidence of high prevalence of STH infections, confirming that STH constitute a public health problem in the states of Chhattisgarh, Telangana, and Tripura. In high STH prevalence settings, augmenting the deworming programs with integrated water, sanitation, and hygiene (WASH) activities is necessary for STH control. The Government of India has initiated biannual mass deworming program in all states. The prevalence estimates from the survey would serve as baseline for documenting the impact of the NDD program in these states in future years.
